# Beyond rigid docking: deep learning approaches for fully flexible protein–ligand interactions

**DOI:** 10.1093/bib/bbaf454

**Published:** 2025-09-03

**Authors:** John Lee, Canh Hao Nguyen, Hiroshi Mamitsuka

**Affiliations:** Bioinformatics Center, Institute for Chemical Research, Kyoto University, Uji 611-0011, Japan; Bioinformatics Center, Institute for Chemical Research, Kyoto University, Uji 611-0011, Japan; Bioinformatics Center, Institute for Chemical Research, Kyoto University, Uji 611-0011, Japan

**Keywords:** flexible docking, molecular docking, protein–ligand interaction, diffusion models, co-folding

## Abstract

Sparked by AlphaFold2’s groundbreaking success in protein structure prediction, recent years have seen a surge of interest in developing deep learning (DL) models for molecular docking. Molecular docking is a computational approach for predicting how proteins interact with small molecules known as ligands. It has become an essential tool in drug discovery, enabling structure-based virtual screening (VS) methods to efficiently explore vast libraries of drug-like molecules and identify potential therapeutic candidates. However, traditional docking methods primarily rely on search-and-score algorithms, which are computationally demanding. To be viable for VS applications, these methods often sacrifice accuracy for speed by simplifying their search algorithms and scoring functions. Recent advancements in DL have transformed molecular docking, offering accuracy that rivals—or even surpasses—traditional approaches while significantly reducing computational costs. Despite these advancements, DL-based molecular docking still faces major challenges. DL models often struggle to generalize beyond their training data and frequently mispredict key molecular properties, such as stereochemistry, bond lengths, and steric interactions, leading to physically unrealistic predictions. To overcome these limitations, a new generation of models is using DL to incorporate protein flexibility into docking predictions, aiming to more accurately capture the dynamic nature of biomolecular interactions—a long-standing challenge for traditional methods. This review explores how DL has reshaped molecular docking, examines its current shortcomings, and highlights emerging solutions. Finally, we discuss future opportunities to further bridge the gap between computational predictions and real-world molecular interactions.

## Introduction

The development of novel therapeutics is a lengthy and costly endeavor, typically spanning 12–15 years with costs exceeding $1 billion USD [1]. A cornerstone of drug discovery relies on identifying and designing ligands that target key proteins involved in disease pathways. In an effort to accelerate this process, computational techniques such as structure-based virtual screening (VS) have emerged as powerful tools, allowing researchers to evaluate large libraries of drug-like molecules *in silico*. VS has become increasingly popular in the field of drug discovery as *in silico* techniques are much faster and cheaper than traditional *in vitro* approaches. Additionally, new computational methods are continuously being developed and improved for accuracy, speed, and reliability [2]. Molecular docking is a key component of VS; it predicts the binding conformations and affinities of protein–ligand complexes, making it the primary approach when the 3D-structure of a target protein is available [3]. As advances in structural biology now allow for the rapid and accurate generation of 3D protein structures [4], further development of molecular docking tools has become increasingly important.

Traditional docking approaches, first introduced in the 1980s [3, 5], primarily follow a search-and-score framework, exploring a vast space of possible ligand poses and predicting optimal binding conformations based on scoring functions that estimate protein–ligand binding strength. These methods are often computationally demanding due to the high dimensionality of the conformational space for both the ligand and the protein. Early methods addressed this challenge by treating both the ligand and protein as rigid bodies, reducing the degrees of freedom to six (three translational and three rotational) [3, 6]. Although this significantly improved computational efficiency, the rigid docking assumption oversimplifies the binding process since, in reality, both ligands and proteins undergo dynamic conformational changes upon interaction. Consequently, these early models often perform poorly in many cases and fail to generalize across different docking tasks, making them less suitable for large-scale VS. To balance computational efficiency with accuracy, most modern molecular docking approaches allow ligand flexibility while keeping the protein rigid [7, 8]. However, modeling receptor flexibility remains crucial for accurately and reliably predicting ligand binding, yet it remains a challenge for traditional methods. This difficulty arises from the exponential growth of the search space and the limitations of conventional scoring algorithms, which are not designed to accommodate protein flexibility.

**Figure 1:**
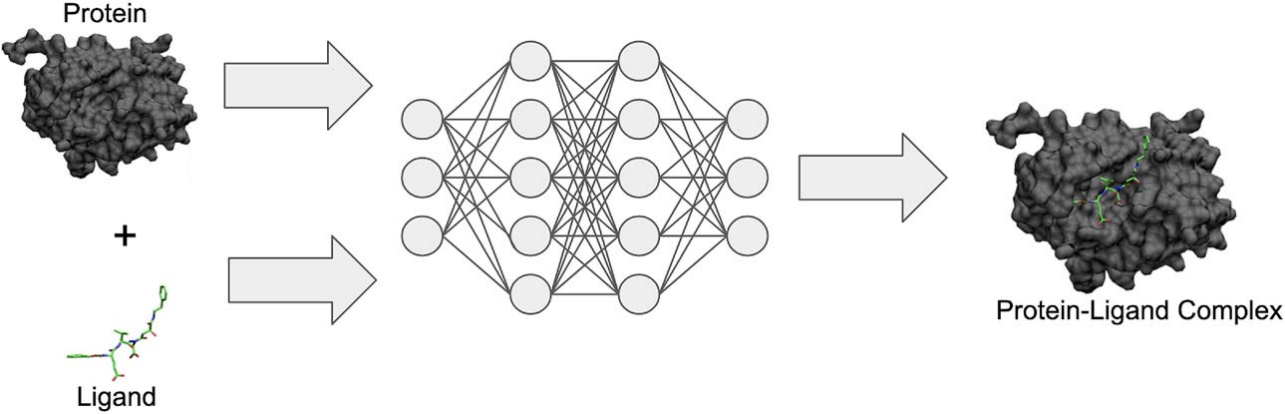
Predicting protein–ligand complexes from protein and ligand pairs using deep learning. Figure adapted from [12].

In recent years, deep learning (DL) models for predicting protein–ligand binding structure (see Fig. 1) have rapidly transformed the field of molecular docking. In 2022, Stärk *et al*. [9] developed EquiBind, an equivariant graph neural network (EGNN) based approach for predicting the 3D structure of protein–ligand complexes. Their method utilized an EGNN to identify “key points” on both the ligand and protein, then used the Kabsch algorithm to find the optimal rotation matrix that minimizes the root mean squared deviation (RMSD) between the two sets of key points. This approach was quickly followed by TankBind [10], which instead opted to use a trigonometry-aware GNN method to predict a distance matrix between protein residues and ligand atoms, as well as a binding affinity score. They then deployed a multi-dimensional scaling method to reconstruct the 3D structure of the protein–ligand complex from the distance matrix. Although transformative, these early DL based models had clear limitations—most notably, they failed to outperform classical docking methods [11], and often predicted physically implausible complexes, i.e. improper bond angles and lengths.

To address these challenges, Corso *et al*. [13] developed DiffDock, which introduced diffusion models to molecular docking. To train their model, Corso *et al*. [13] used experimentally determined protein–ligand complexes from PDBBind, and progressively added noise to the ligand’s degrees of freedom (translation, rotation, and torsion angles). An SE(3)-EGNN then learns a denoising score function to iteratively refine the ligand’s pose back to a plausible binding configuration. After training, the model predicts complex structures for previously unseen protein–ligand inputs. Corso *et al*. [13] demonstrated that DiffDock achieved state-of-the-art accuracy on a PDBBind test set, while operating at a fraction of the computational cost compared with traditional methods. Additionally, DiffDock predicted more physically plausible structures when compared to previous DL methods (i.e. EquiBind and TankBind).

Early DL docking methods, such as EquiBind and DiffDock, drew criticism for being unfairly compared to traditional approaches. Unlike conventional methods that assume the binding pocket is known, these models perform blind docking—a harder task for which classical algorithms are not designed for. To enable fairer comparison, Yu *et al*. [11] separated blind docking into pocket identification and ligand docking. They found that DL models outperform traditional methods in identifying pockets, but underperform when docking into known pockets. This suggests DL models may focus more on locating binding sites than on accurate pose prediction. A similar issue was noted in the EquiBind study, leading to a hybrid proposal: use DL to predict the binding site, then refine poses with conventional docking. Despite these limitations, DL-based docking remains a promising and rapidly evolving field.

Before their application in structure prediction of protein–ligand complexes, machine learning techniques have been widely used to enhance scoring functions in traditional docking. These enhancements enabled faster and more scalable predictions of binding affinities compared to conventional energy-based calculations. Notable graph neural network (GNN) based approaches include IGN [14], GIGN [15], and PIGNet [16]. These methods employ GNNs to predict stability or binding affinity of potential protein–ligand complexes. They then rank these complexes based on the likelihood of their predicted interactions reflecting true biological interactions. However, since this review focuses on DL methods for structure prediction rather than binding affinity prediction, we do not explore these methods in detail. For a comprehensive overview of recent advancements in DL-based scoring functions, we direct readers to existing review articles [17].

### Protein flexibility

The majority of DL approaches follow in the footsteps of traditional docking methods by only accommodating ligand flexibility while largely treating the protein receptor as rigid. This oversimplification presents significant challenges in real-world scenarios, such as cross-docking, apo-docking, or cases involving computationally predicted protein structures. Table 1 summarizes common docking tasks within the field of MD. These challenges arise from the fact that proteins are inherently flexible and can undergo substantial conformational changes upon ligand binding—a phenomenon known as the *induced fit effect*. As a result, the binding pocket of an apo structure may differ significantly from its ligand-bound (holo) counterpart. Without accounting for these induced fit effects, docking methods trained primarily on holo structures, such as those in PDBBind, struggle to accurately predict binding poses when docking to apo conformations. Addressing this issue has driven recent advances in DL-based docking that incorporate protein flexibility.

**Table 1:** Overview of protein–ligand docking tasks, which evaluate molecular docking models across varying degrees of structural uncertainty and practical relevance to test their robustness, generalization, and applicability in drug discovery pipelines.

Docking task	Description
**Re-docking**	Involves docking a ligand back into the bound (holo) conformation of the receptor. This task evaluates whether a model can recover the original binding pose from an idealized protein–ligand complex. DL models trained on datasets like PDBBind typically perform well here, but may overfit to the ideal geometries, limiting their ability to generalize to unseen or non-ideal cases.
**Flexible re-docking**	Uses holo structures with randomized binding-site sidechains to introduce local perturbations. Evaluates model robustness to minor conformational changes.
**Cross-docking**	Ligands are docked to alternative receptor conformations (e.g. from different ligand complexes). This is illustrated in Fig. 2. This simulates real-world cases where ligands are docked to proteins in unknown conformational states.
**Apo-docking**	Uses unbound (apo) receptor structures, typically obtained from crystal structures or computational predictions. This is a highly realistic setting for drug discovery, requiring models to infer the induced fit and accommodate structural differences between the unbound and bound states.
**Blind docking**	Requires prediction of both the ligand pose and the binding site location. It is the most challenging and least constrained task. However, it is less common in practical settings where binding sites are often known.

**Figure 2:**
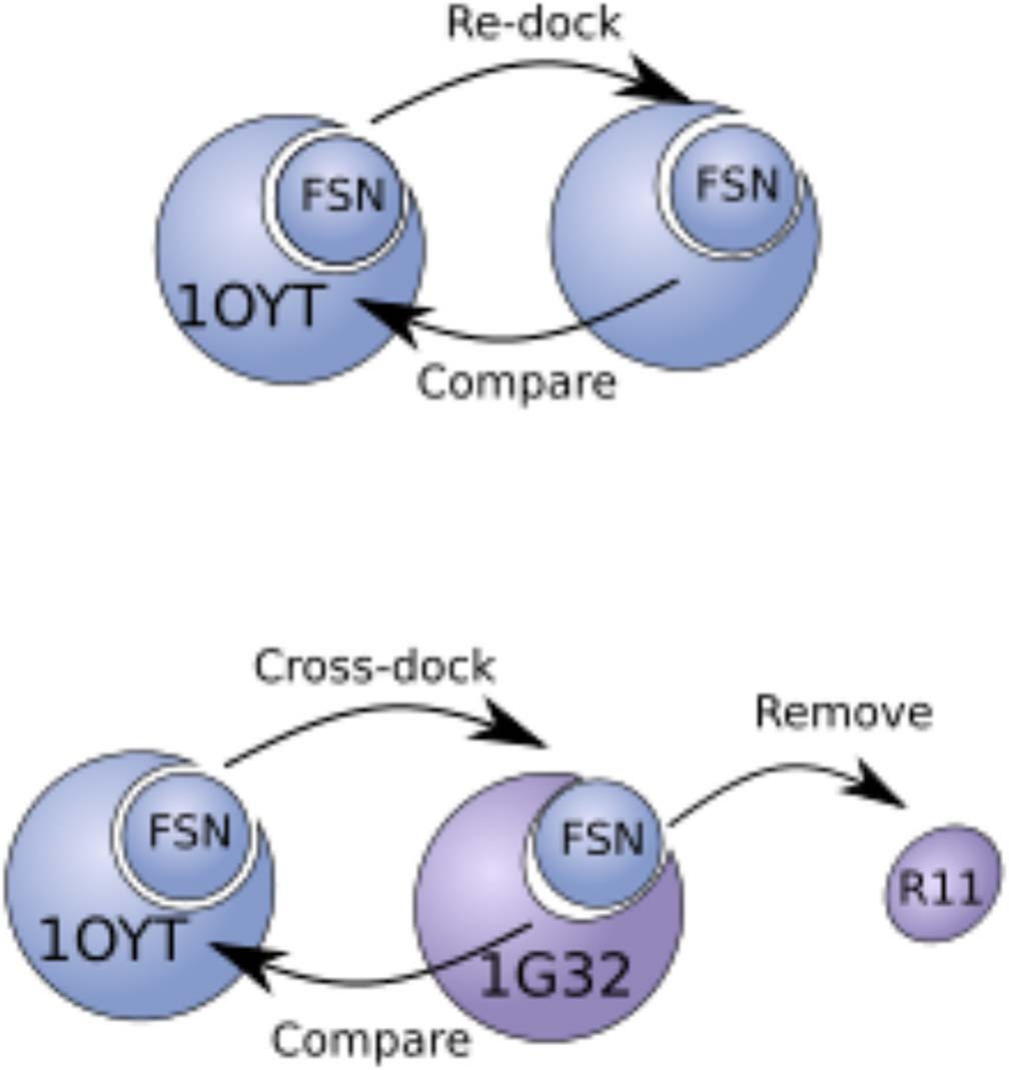
Visual illustration of re-docking versus cross-docking. Figure adapted from [18].

Conformational changes during docking are often small and localized, largely due to ligand size and energetic constraint. A key component of flexible docking methods is the ability to predict how an unbound (apo) protein undergoes conformational changes to adopt its bound (holo) state upon ligand binding. Because conformational changes tend to occur near the binding site, accurately capturing sidechain flexibility is especially important in docking, as it directly impacts pose prediction performance [19]. Early diffusion-based methods, such as DiffDock, partially accounted for protein flexibility by coarsely representing protein structures, allowing small conformational adjustments at the residue level within the binding pocket. While this reduced sensitivity to atomic misplacements and mitigated the need for exhaustive sampling, it still treated flexibility in an indirect manner, limiting its ability to handle significant conformational rearrangements.

Building on these efforts, Chen *et al*. [20] developed FlexPose, enabling end-to-end flexible modeling of the 3D structure of protein–ligand complexes irrespective of input protein conformation (apo or holo). Similarly, aligned diffusion Schrödinger Bridges [21] have been proposed as a method to predict conformational transitions between apo and holo states by treating them as paired data, offering a novel framework for modeling protein flexibility. However, this method has yet to be evaluated for flexible protein–ligand docking, leaving it as an open avenue for future research.

Another emerging direction focuses on identifying cryptic pockets—transient binding sites hidden in static structures but revealed through protein dynamics. Methods like DynamicBind [22] are capable of revealing these cryptic pockets by using equivariant geometric diffusion networks to model protein backbone and sidechain flexibility. Notably, they do so at a fraction of the computational cost of traditional molecular dynamics simulations and turn previously “undruggable” proteins into potential drug targets. In a compelling case study involving the histone methyltransferase SETD2, DynamicBind accurately predicted the binding pose of EZM0414, a selective inhibitor currently in Phase I clinical trials, targeting a cryptic pocket that was unseen in any training structure. The predicted pose achieved a ligand RMSD of 1.4 Å, despite the absence of similar ligands or pocket conformations in training data. These results underscore the promise of flexibility-aware DL methods for discovering novel druggable sites and improving docking in challenging settings like cross-docking and apo-docking.

By modeling protein flexibility, these approaches move beyond the rigid-receptor paradigm, enabling more realistic and robust docking predictions. This review provides a comprehensive overview of emerging models and techniques that account for protein flexibility, serving as a valuable resource for researchers exploring the latest advances at the intersection of DL and molecular docking.

## Criticisms

In 2023, early DL methods like EquiBind, TankBind, and DiffDock underwent further testing to assess their reported performance. Two key criticisms emerged:

- Overfitting to training datasets that lead to an inability to generalize.
- A tendency to generate physically implausible structures.

### PoseBusters and the problem of generalizability

Many DL docking models—including DiffDock, DeepDock, EquiBind, TankBind, and Uni-Mol—are trained on datasets composed of experimentally resolved protein–ligand complexes, most commonly PDBBind. However, because PDBBind exclusively comprises experimentally determined structures, its composition is inherently biased toward protein families with extensive structural characterization. For instance, kinases constitute a disproportionately large portion of the dataset, whereas G protein–coupled receptors (GPCRs) and ion channels are underrepresented, with the latter accounting for ¡5% of entries. This underrepresentation arises from experimental challenges in resolving the 3D structures of both protein families. Moreover, PDBBind suffers from significant data leakage due to high sequence and chemical similarity between training and test sets, especially within the commonly used “core set,” which contains smaller ligands and better-resolved structures but overlaps heavily with training data. This issue is also evident in benchmarks like the Astex Diverse set, where a significant proportion of test complexes resemble those seen during training. This inflates performance metrics and masks poor generalization to novel targets.

To quantify and highlight the impact of such similarity-induced bias, some studies [23] have adopted sequence-based and ligand scaffold-based splits, which reveal a substantial drop in performance, confirming that similarity biases strongly affect model predictions. Similarly, time-based splits have been proposed as a practical compromise to mitigate leakage [9, 24]. In this approach, models are trained only on complexes released before a cutoff year (e.g. 2019) and evaluated on newer entries (after 2019). While this mimics real-world deployment more closely and helps reduce overlap, it does not fully resolve the problem as newly tested compounds often target previously studied proteins, and vice versa, resulting in persistent similarity between training and test sets.

The performance of these models further declines when evaluated on deliberately curated, out-of-distribution datasets. The PoseBusters benchmark set [25], for example, was designed to assess the generalization capabilities of DL models, includes protein–ligand complexes released after 2021 and ensuring they are absent from standard training sets like PDBBind 2020. This benchmark revealed that all tested DL models produced higher RMSD values on average, highlighting their struggle to generalize beyond their training data. In contrast, classical docking approaches, such as AutoDock Vina and GOLD, demonstrated more consistent performance across standard evaluation metrics.

These findings suggest that while DL-based methods can capture certain geometric features under favorable conditions, they lack the inductive biases necessary for robust generalization across the broader chemical and biological space encountered in real-world applications.

### The problem of physically implausible predictions

Beyond generalization issues, DL-based docking methods often generate physically implausible ligand poses that look accurate based on RMSD scores but break basic physical and chemical rules. The widespread reliance on RMSD as a primary evaluation metric has masked these deficiencies, as a low RMSD relative to a crystallographic pose does not guarantee physical plausibility. Table 2 summarizes some commonly used metrics for evaluating protein–ligand docking models.

**Table 2:** Summary of protein–ligand docking evaluation metrics.

Metric	Description
RMSD $\leq $ 2 Å	Measures the average distance between atoms of the predicted and experimental ligand poses. An RMSD of 2 Åor less is typically considered a successful prediction. Models are typically assessed on the percentage of predicted structures which have RMSD $\leq $ 2 Å.
RMSD $\leq $ 2 Å& PB-Valid	Combines the RMSD threshold with validation checks from the PoseBusters (PB) software suite. A pose is successful if it has an RMSD $\leq $ 2 Åand passes PB-Valid checks, which include assessments of chemical and physical plausibility.
lDDT-PLI	Evaluates the accuracy of predicted protein–ligand interactions by comparing the local atomic environments of the predicted and experimental structures. Higher lDDT-PLI scores indicate better agreement with the reference, focusing on the quality of the binding interface.
PLIF-EMD	Assesses how well the predicted protein–ligand interaction patterns match the experimental data by calculating the Earth Mover’s Distance between predicted and actual interaction fingerprints. Lower PLIF-EMD values indicate a closer match to the native interaction patterns.
BiSyRMSD	Measures the absolute deviation of a predicted ligand pose from the experimentally determined structure, focusing on the binding site and accounting for any symmetry in the ligand to provide a more precise assessment of docking accuracy.

Benchmark studies such as PoseBusters [25] found that over 50% of ligand poses generated by DL models failed stringent validity checks, exhibiting critical errors such as incorrect stereochemistry, unrealistic bond lengths and angles, and severe steric clashes with the protein receptor. These errors introduce significant strain energies, making such predictions unlikely to correspond to biologically relevant binding modes. In contrast, classical docking methods such as AutoDock Vina and CCDC GOLD produced invalid structures in only 2%–3% of cases, demonstrating a superior ability to enforce physical constraints.

These findings indicate that early DL docking models often miss important biophysical constraints needed for accurate predictions. In the next sections, we will discuss the architectural innovations that lead to the surge of DL-based approaches for molecular docking before discussing new advancements that attempt to resolve the criticisms mentioned above.

## Architectural innovations

GNNs have had a profound impact on molecular docking, from their early adoption in DL-based scoring functions like IGN and PIGNET to their central role in structure prediction models such as EquiBind and DiffDock. Their success stems from their ability to process graph-structured data, making them well-suited for modeling proteins, ligands, and their interactions.

### Ligand and protein graph representations

Ligands are typically modeled as molecular graphs, where nodes represent atoms and edges represent chemical bonds or spatial proximity (e.g. $k$-nearest neighbor graphs [9]). Each node carries a feature vector encoding atomic properties such as element type, valence, and chirality, along with 3D coordinates to preserve spatial context. Edges encode bond-specific features like type, length, and directionality—often computed using cheminformatics toolkits like RDKit.

Proteins can also be represented as graphs, generally at one of two resolution levels: atomic (fine-grained) or residue (coarse-grained). Coarse-grained graphs are common in recent models due to their ability to capture higher-order structural features, such as backbone orientation, while significantly reducing graph size and improving computational efficiency. As with ligands, protein graphs include rich node and edge features along with spatial coordinates.

To model protein–ligand interactions, DL models construct intermolecular graphs that integrate binding site information. For example, EquiBind connects nodes between the protein graph ($G_{p}$) and the ligand graph ($G_{l}$) by edges, enabling the model to identify key binding interactions between the two structures. A more exhaustive approach, KarmaDock [26], creates a fully connected intermolecular graph, linking every node in $G_{l}$ to every node in $G_{p}$. While comprehensive, this method is computationally expensive due to quadratic edge scaling. To balance accuracy and efficiency, DiffDock employs KNN-based intermolecular graphs, connecting only spatially proximal protein and ligand nodes. This strategy reduces computational complexity while preserving key geometric and chemical constraints for accurate binding predictions.

### Graph neural networks

In a GNN each node of an input graph is first initialized with a feature vector $\mathbf{h}_{i}^{(0)}$ which is then updated iteratively through message passing. At each layer $l$, every node $v_{i}$ aggregates messages from its neighbors $N(i)$ using a learnable message function $\psi $, which computes 


\begin{align*} & \mathbf{m}_{i}^{(l)} = \bigoplus_{j \in N(i)} \psi\big(\mathbf{h}_{i}^{(l)}, \mathbf{h}_{j}^{(l)}, \mathbf{e}_{ij}\big), \end{align*}$$

where $\bigoplus $ denotes a permutation-invariant aggregation operation (such as summation or averaging) and $\psi $ includes trainable parameters (e.g. weights of a feedforward neural network). The node then updates its representation via another learnable function $\phi $ such that 


\begin{align*} & \mathbf{h}_{i}^{(l+1)} = \phi\big(\mathbf{h}_{i}^{(l)}, \mathbf{m}_{i}^{(l)}\big), \end{align*}$$

i.e. the node representation is updated using a learnable function which takes as input the node representation from the previous layer, and the “message” from its neighbors. After several message-passing layers, each node’s representation encodes information from an increasingly large neighborhood, capturing both local and global graph structure. For graph-level tasks, a readout function $\rho $ aggregates the final node embeddings $\{\mathbf{h}_{i}^{(L)}\}$ into a fixed-size representation, 


\begin{align*} & \mathbf{h}_{G} = \rho\big(\{\mathbf{h}_{i}^{(L)}: i \in V\}\big), \end{align*}$$

which is then fed into further learnable layers to yield the final prediction.

#### Equivariant graph neural networks

**Figure 3:**
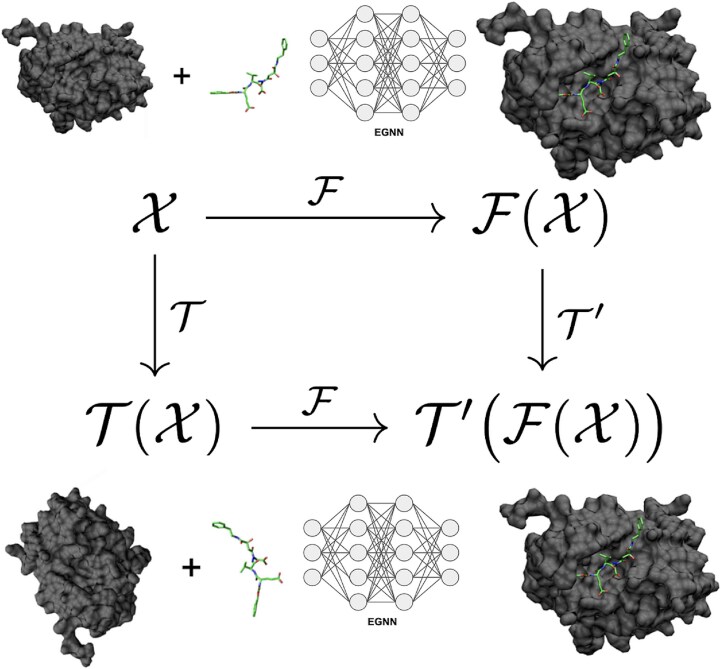
Illustration of the equivariance property in neural networks, where transformations applied to the input (e.g. rotation or translation) produce corresponding transformations in the output, ensuring consistent predictions regardless of molecular orientation.

Equivariance is a critical property for models that predict 3D protein–ligand complex structures. As illustrated in Fig. 3, it ensures that a transformation applied to the input (e.g. a rotation or translation) leads to a predictable transformation of the output. Formally, a neural network $ \mathcal{F} $ is equivariant to a transformation $ \mathcal{T} $ if, for any input $\mathcal{X}$ and some corresponding transformation $ \mathcal{T}^{\prime} $ in the output space, it holds that 


\begin{align*} & \mathcal{F}(\mathcal{T}(\mathcal{X})) = \mathcal{T}^{\prime}\big(\mathcal{F}(\mathcal{X})\big). \end{align*}$$

This means that every layer in the network must transform its features in a way that respects the symmetry of the underlying physical system—typically the special Euclidean group $ \textrm{SE}(3) $ for 3D space. In molecular docking, such equivariance guarantees that the predicted complex structure remains consistent regardless of arbitrary rotations or translations of the input molecules. For example, EquiBind, enforces $ \textrm{SE}(3) $ equivariance by conditioning its message passing on the invariant squared distance $||\mathbf{x}_{i}^{(l)} - \mathbf{x}_{j}^{(l)}||^{2}$, ensuring that messages 


\begin{align*} & \psi\big(\mathbf{h}_{i}^{(l)}, \mathbf{h}_{j}^{(l)}, \|\mathbf{x}_{i}^{(l)} - \mathbf{x}_{j}^{(l)}\|^{2}\big) \end{align*}$$

remains unchanged under rotations and translations. While this approach is simple and data-efficient—often outperforming non-equivariant models with less training data—it may not fully capture richer geometric details. As a result, more advanced methods, such as those used in DiffDock, incorporate group-theoretic frameworks by representing node and edge features as irreducible representations via spherical harmonics. These representations are combined through operations like the Clebsch–Gordon tensor product to rigorously maintain $ \textrm{E}(3) $ equivariance throughout the network while allowing the model to learn complex, informative geometric relationships. Although EGNNs are generally more challenging to design and scale when compared to their non-equivariant counterparts, practitioners benefit from the **e3nn** [27] library—a widely used PyTorch-based tool that simplifies the implementation and development of EGNNs.

#### Is equivariance all you need?

Recent studies have questioned whether hard-coded equivariance is essential for effective DL–based molecular docking [28–30]. With sufficient training data, some argue that it’s more practical to use scalable architectures that learn symmetries via data augmentation, rather than enforcing them explicitly—an approach exemplified by models like AlphaFold3 and Chai-1, which prioritize scalability over strict equivariance.

However, for smaller, specialized models—such as those focused on protein–ligand docking—equivariance remains relevant. Unlike large biomolecular networks, designed for scalability, these models can leverage explicit symmetry constraints to enhance accuracy and generalization, particularly in data-scarce settings. Equivariance ensures rotational and translational consistency without excessive data augmentation, making it a principled approach to enforcing biophysical constraints.

Despite these advantages, there are practical challenges. Equivariant models often introduce computational overhead and are challenging to scale. A hybrid approach may offer the best balance, combining equivariance to capture fundamental symmetries with data augmentation to model complex, emergent behaviors. Future research comparing these strategies on protein–ligand interaction datasets will be key to determining the optimal approach.

### Transformers

Transformer-based models have advanced protein–ligand interaction prediction by capturing long-range dependencies via self-attention mechanisms. Uni-Mol [31] introduced pair-biased attention to jointly encode atom- and pair-level features, predicting ligand atom coordinates from pairwise distance matrices. Building on this, GAABind [32] incorporated triangular attention, inspired by AlphaFold2, to better capture the geometric structure of binding pockets.

Despite their strengths, transformer models typically require large training datasets and rely on separate pose generation and scoring steps, which can lead to high computational costs and physically implausible conformations (e.g. steric clashes). CarsiDock [33] addresses these limitations through large-scale pretraining followed by fine-tuning on crystallized complexes to improve generalization.

More recently, Interformer [34] introduced a unified Graph-Transformer architecture that models both local and global interactions using specialized intra- and inter-molecular attention blocks. It incorporates a mixture density network to explicitly model non-covalent interactions (e.g. hydrogen bonds, hydrophobic forces) and uses contrastive learning to improve pose sensitivity, generating more physically realistic docking predictions.

### Diffusion models

Diffusion models have recently emerged as a powerful tool in computational structural biology, enabling more accurate and scalable modeling of molecular structures. Earlier approaches such as variational autoencoders (VAEs) and generative adversarial networks (GANs) often struggled to handle the geometric constraints of molecules or scale effectively to their high-dimensional representations [35]. In contrast, diffusion models offer a robust framework to learn complex structural distributions, leading to significant advances in tasks like protein design, molecular docking [24], and biomolecular interaction prediction [36].

**Figure 4:**
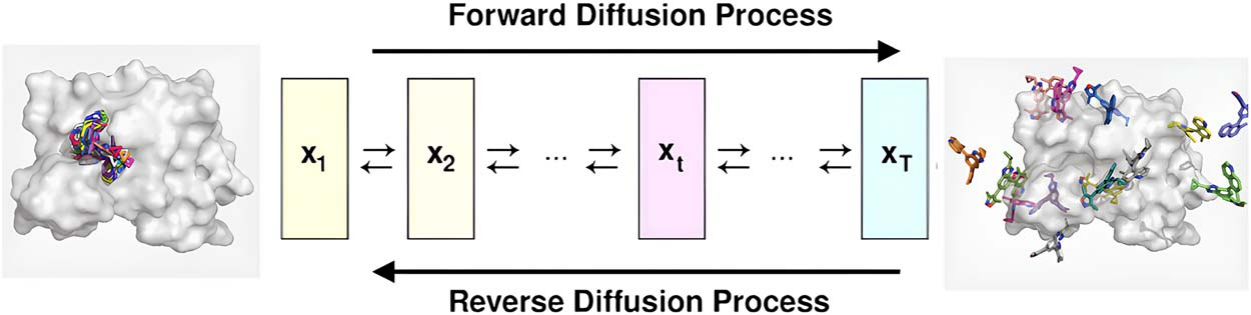
Fixed protein diffusion process, where ligands from experimentally determined complexes are progressively noised with random translations, rotations, and torsional perturbations, and a diffusion model is trained to reverse this process to denoise and redock ligands into the correct binding pocket (adapted from [24]).

Score-based diffusion models, one of the earliest formulations of diffusion modeling, operate by learning to reverse a stochastic process that incrementally degrades data into noise, as illustrated in Fig. 4. The forward process transforms a data sample $ x_{0} \sim p_{0} $ into Gaussian noise $ x_\tau \sim p_\tau $ via a stochastic differential equation (SDE): 


(1) \begin{align*}& dx = f(x,t) dt + g(t) dB\end{align*}$$

where $ f(x,t) $ is the drift term, and $ g(t) dB $ injects Gaussian noise through Brownian motion. The diffusion model is then trained to reverse this process, by approximating another SDE which transforms Gaussian noise to samples from the data distribution: 


(2) \begin{align*}& dx = \left[f(x,t) - g(t)^{2} \nabla \log p_{t}(x) \right] dt + g(t) dB\end{align*}$$

where $ \nabla \log p_{t}(x) $ (the Stein score) guides samples toward high-probability regions. Since $ p_{t}(x) $ is intractable, the score function is approximated by a neural network $ s_\theta (x,t) $, trained via denoising score matching: 


(3) \begin{align*}& L = \mathbb{E}_{x_{0} \sim p_{0}, x_{t} \sim p_{t|0}} \left[ \|s_\theta(x,t) - \nabla \log p_{t|0}(x|x_{0})\|^{2} \right]\end{align*}$$

where $ p_{t|0}(x | x_{0}) $ is an analytically tractable conditional distribution. Sampling is performed by discretizing the reverse SDE using Euler–Maruyama integration.

#### Equivariance in diffusion models

Equivariance is a crucial property in diffusion models applied to molecular systems. Since molecular structures should maintain the same probability distribution regardless of their orientation in 3D space, diffusion models must be invariant to translations and rotations. To ensure translation invariance, molecular structures are zero-centered by subtracting the center of mass during training and inference. Rotation invariance is achieved if the diffusion model’s score function is equivariant, which can be implemented using an EGNN to approximate the score function. Selecting an appropriate neural network architecture is essential for ensuring that diffusion models generalize well and generate physically meaningful molecular structures.

#### Future of diffusion models

Researchers are actively exploring robust generalizations to score based diffusion models such as flow matching and Schrödinger bridges to further enhance diffusion-based generative modeling. Flow Matching, for example, learns a vector field which induces a continuous normalizing flow that transports a source distribution to a target distribution. These advancements balance stochastic and deterministic elements in the modeling process, offering flexible prior distributions without requiring explicit formulations. As the field evolves, diffusion models, and their spin-offs, are poised to remain fundamental tools in structural biology, continually improving our understanding of protein–ligand interactions.

In later sections, we discuss how diffusion-based approaches have been utilized to model protein flexibility, leading to improved performance and accuracy on challenging cross-docking tasks and apo docking scenarios. By modeling flexibility, these methods generate more physically realistic binding poses and better generalize to unseen protein conformations.

## Flexible methods

The evolution of molecular docking programs is characterized by models which fall into three categories:

- **Fully rigid docking**: Both proteins and ligands are treated as rigid structures.
- **Ligand flexible methods**: Proteins are assumed to be rigid while ligands are flexible.
- **Protein flexible methods**: Both the ligand and protein are flexible.

In recent years, growing interest in accounting for protein conformational changes induced by ligand docking has lead to the emergence of two promising strategies:

- **Flexible docking** methods model the structural transition from the apo to the holo state of a protein, enabling docking to conformations that better reflect induced-fit effects.
- **Co-folding** methods model the structure of protein–ligand complexes directly from input data (typically sequences) in a single task, inherently modeling induced-fit effects.

In the following sections, we review recent advancements in protein flexible DL molecular docking methods, evaluating their strengths and limitations.

### Flexible docking

Flexible docking methods predict the conformational changes proteins undergo upon ligand binding. Some traditional search-based docking methods incorporate protein flexibility by adding sidechain torsions to the search space [37]. However, this significantly increases dimensionality, making it challenging to identify optimal binding poses. Nonetheless, the importance of incorporating protein flexibility has been understood for decades. For example, Zavodsky and Kuhn’s 2005 study [38], found that while rigid docking failed in half of the cases, accounting for sidechain flexibility enabled the successful re-docking of all 63 ligands in their study.

Conformational changes during docking are often small and localized, largely due to ligand size and energetic constraints. As a result, many flexible docking methods focus on sidechain flexibility, though some recent approaches also model backbone flexibility [22, 39, 40] to capture larger conformal shifts. These methods are typically more scalable than traditional sampling techniques or DL–based co-folding approaches, which can take minutes per prediction. Flexible docking methods generally fall into two main categories:

- **Implicitly** flexible models account for flexibility by adjusting scoring functions or using coarse representations to partially account for potential conformational changes. By implicitly considering protein flexibility, these methods improve docking accuracy without incurring the computational cost of explicit simulations.
- **Explicitly** flexible models directly predict the changes to atomic coordinates of proteins during the docking process. Techniques such as flexible sidechain modeling allow for a more realistic representation of the protein’s conformational space, leading to more accurate predictions of ligand binding.

A summary of recent DL-based molecular docking models, including their architectures, handling of protein flexibility, and input representations, can be found in Table 3, and benchmark performance of a handful of these methods can be found in Table 4.

**Table 3:** Prominent deep learning models for molecular docking, categorized by architectural features (e.g. GNNs, diffusion models, transformers, equivariant mechanisms), treatment of protein flexibility (fixed, implicit, explicit), and input representations (sequence-based, structure-based, hybrid).

Models	Architecture				Protein Flexibility				Input Representation		
	GNN	Diffusion	Transformer	Equivariant	Fixed	Implicit	Explicit	Co-folding	Sequence	Structure	Hybrid
EquiBind [9]	✓			✓	✓					✓	
TankBind [10]	✓			✓	✓					✓	
DiffDock [13]	✓	✓		✓	✓					✓	
KarmaDock [26]	✓		✓	✓	✓					✓	
GAABind [32]			✓		✓					✓	
Interformer [34]	✓		✓		✓					✓	
CarsiDock [33]			✓	✓	✓					✓	
SurfDock [42]	✓	✓		✓		✓					✓
EDM-Dock [43]	✓		✓	✓		✓				✓	
FLEXVDW [41]	✓		✓	✓		✓				✓	
QuickBind [44]			✓	✓		✓					✓
DiffDock-Pocket [19]	✓	✓		✓			✓			✓	
FlexPose [20]	✓		✓	✓			✓			✓	
DynamicBind [22]	✓	✓		✓			✓			✓	
FlexDock [45]	✓	✓		✓			✓			✓	
Re-Dock [46]	✓	✓		✓			✓			✓	
FlexiDock [39]	✓	✓		✓			✓			✓	
CarsiDock-Flex [47]			✓	✓			✓			✓	
ApoDock [40]	✓		✓				✓			✓	
FlowDock [48]		✓		✓			✓		✓		
DiffBindFR [39]	✓	✓		✓			✓			✓	
UMOL [49]			✓				✓		✓		
RoseTTAFold All-Atom [50]			✓					✓			✓
AlphaFold3 [36]		✓	✓					✓	✓		
Chai-1 [51]		✓	✓					✓	✓		
Boltz-1 [52]		✓	✓					✓	✓		
NeuralPLexer3 [53]		✓	✓	✓				✓			✓

**Table 4:** Comparison of selected docking models, showing success rate (ligand RMSD < 2 Å) on the PoseBusters Benchmark, PB validity (successful dockings passing physical plausibility checks), and flexibility method categorized as explicit (E), implicit (I), or co-folding (C).

Model	Flexibility	Success rate (%)	PB validity (%)	Flexibility method
EquiBind [9]	$\times $	2.0	0.0	–
DiffDock [24]	$\times $	38	12	–
CarsiDock [33]	$\times $	79.7	47.7	–
Re-Dock [46]	✓	50.7	32.8	E
DiffBindFR-Smina [56]	✓	50.2	49.1	E
DynamicBind [22]	✓	67.8	34.0	E
FlowDock [48]	✓	64.5	37.5	E
SurfDock [42]	✓	82	74	I
AlphaFold3 [36]	✓	80.4	73.1	C
NeuralPLexer3 [53]	✓	80.2	77.9	C

### Implicit flexibility

#### Atomic and residue-level representations

Implicit flexibility methods aim to capture conformal changes without explicitly sampling multiple receptor states, offering a compelling tradeoff between accuracy and efficiency. FLEXVDW [41] exemplifies this at an atomic level, it implicitly accounts for protein flexibility by refining Van der Waals (VDW) interaction energy predictions without explicit conformational change modeling. It leverages multiple holo structures from PDBBind during training to learn how binding pockets deform across different ligands. At inference time, however, it requires only a single receptor conformation, yet still estimates the most favorable VDW interactions as if multiple conformers were available. The model architecture combines 3D equivariant convolution layers over atomic point clouds with pairwise interaction modules to capture fine-grained spatial relationships between ligand and receptor atoms. Integrated into the Glide docking suite, FLEXVDW improves pose prediction accuracy for targets with substantial flexibility while maintaining performance on rigid systems. Trained on the PDBBind 2019 refined set and evaluated on 615 cross-docked pairs, FLEXVDW demonstrates significant gains in challenging flexible cases without compromising efficiency.

EDM-Dock [43] builds on a similar philosophy of flexibility through abstraction, using an EGNN to predict Euclidean distance matrices (EDMs) between ligand atoms and the protein’s coarse-grained C$\alpha $ “skeleton.” Unlike FLEXVDW, which operates on full-atom input, EDM-Dock frees sidechains during pose generation, allowing them to respond dynamically to the ligand via an energy minimization step that lightly restrains the backbone. This enables local flexibility near the binding site without needing a full receptor ensemble. Trained on 53 000 BioLiP complexes, EDM-Dock outperforms GeauxDock by 0.77Å in redocking RMSD and shows stronger cross-docking performance than both GeauxDock and AutoDock Vina, achieving up to 2.00Å improvements and more frequent near-native poses (within 2Å–3.5Å). Together, FLEXVDW and EDM-Dock illustrate how implicitly flexible representations—whether through multiple training conformers or coarse-grained distance predictions—can improve docking accuracy while remaining computationally tractable.

#### Surface representations

Surface-based representations offer an alternative strategy for modeling protein flexibility by abstracting away atomic-level rigidity while retaining key geometric and chemical features relevant to binding. A foundational method in this space is MaSIF [54], which uses geometric DL to operate directly on protein surfaces. It generates a high-resolution mesh, decomposed into overlapping geodesic patches that capture local properties such as curvature, shape, hydropathy, and electrostatics. These features are processed with geodesic convolutional networks to produce rotation-invariant surface “fingerprints”—compact vector encodings that characterize local binding environments while remaining robust to small conformational changes.

Building on this idea, SurfDock [42] represents the current state-of-the-art among surface-based docking frameworks with implicit protein flexibility. SurfDock integrates MaSIF-derived surface fingerprints with ESM-2 language model embeddings to construct a rich, multimodal representation of the protein’s binding site. This surface-level abstraction not only reduces the need for fixed atomic coordinates but also enables the model to operate within a more physically plausible binding geometry, allowing it to accommodate moderate structural changes without explicitly simulating receptor flexibility. Within an SE(3)-equivariant reverse diffusion framework, SurfDock iteratively refines an initial ligand pose by optimizing rotations, translations, and torsions—guided by the learned surface descriptors. An optional energy minimization step further adjusts ligand positions post-docking, especially in cases where the protein undergoes slight conformational shifts.

Trained on the PDBBind2020 dataset, SurfDock integrates three complementary levels of protein information—sequence, residue graph, and surface geometry—into a unified generative framework. It achieves strong generalization and outperforms both classical and DL-based baselines on a range of benchmarks, including PoseBusters, Astex, and DEKOIS 2.0. Notably, the practical applications of SurfDock are highlighted by its strong performance in VS tasks, particularly on the DEKOIS 2.0 benchmark—a challenging dataset consisting of 81 protein targets, each paired with 40 known actives and 1200 structurally similar decoys designed to test a model’s ability to distinguish true binders. By achieving an enrichment factor (EF0.5%) of 21.00, SurfDock demonstrates its ability to efficiently and reliably prioritize active compounds from large chemical libraries, making it a valuable tool for early-stage hit identification in structure-based drug discovery pipelines.

By leveraging surface geometry as a flexible proxy for binding site conformation, SurfDock exemplifies how DL models can implicitly capture the effects of protein flexibility without requiring explicit modeling.

### Explicit flexibility

DL-based approaches offer a key advantage over traditional methods as they can explicitly model protein flexibility without suffering from intractable computational costs. Notably, DiffDock-Pocket and CarsiDock-Flex represent recent advances in this direction. Both methods extend earlier models by incorporating protein conformational changes into the generative process, significantly improving docking accuracy and enhancing applicability in VS settings.

**DiffDock to DiffDock-Pocket.** In many downstream tasks—such as binding affinity calculations—the accuracy of sidechain conformations is as critical as that of the ligand pose. Recognizing this, DiffDock-Pocket [19] extends DiffDock by incorporating pocket-specific docking and explicit receptor flexibility into a diffusion-based framework. Rather than predicting full atomic coordinates, it models conformational changes in a reduced transformation space defined by ligand translation, rotation, torsion angles, and nearby sidechain torsions. Residues within 3.5Å of the ligand are treated as flexible, with their torsion angles refined through a reverse diffusion process guided by SE(3)-equivariant tensor field networks.

To ensure accurate modeling of flexibility, particularly when using in silico-generated apo protein structures, such as those from ESMFold or ColabFold, DiffDock-Pocket employs a sidechain conformer matching procedure during training. This aligns sidechain torsion angles of predicted structures with experimentally resolved holo structures, while incorporating a steric clash penalty that discourages unrealistic atom overlaps. This approach addresses previous limitations of DL models by improving the physical plausibility of generated complexes. Additionally, it enables the model to maintain strong docking performance even on noisy or unbound structures.

Empirically, DiffDock-Pocket achieves a correct pose (RMSD < 2Å) in 49.8% of cases on the PDBBind benchmark, outperforming prior flexible docking methods, and retains high accuracy for in silico structures (41.7% for ESMFold; 39.5% for ColabFold). The model was also evaluated in cross-docking scenarios, where ligands are docked into alternate protein conformations unseen during training. This metric is particularly relevant when pocket sizes are unevenly distributed—a common challenge in VS. Notably, DiffDock-Pocket out performed prior flexible docking methods despite not being trained on cross-docked structures and using an out-of-distribution pocket definition. When aligned to the training-time pocket definition, its performance improves further, underscoring its strong generalization capabilities. 

However, the precise contribution of receptor flexibility to docking accuracy remains difficult to isolate. While methods like GNINA and DiffDock-Pocket improve physical realism—e.g. by passing more PoseBusters checks—the extent to which these improvements stem from flexible modeling remains unclear. These results underscore the promise of incorporating torsional flexibility but also highlight the need for systematic, large-scale evaluations to fully understand when and how flexibility enhances docking performance.

**CarsiDock to CarsiDock-Flex.** In 2023, Cai *et al*.[33] introduced CarsiDock, a novel DL approach pre-trained on millions of predicted protein–ligand complexes. The model consisted of two main components: a neural network that predicted protein–ligand atomic distance matrices and a geometry optimization step that reconstructed valid binding poses by refining translation, rotation, and torsion. CarsiDock demonstrated strong generalization, achieving a top-1 success rate of 79.7% on the PoseBusters benchmark, outperforming both classical and DL-based docking methods. However, its PB-validity score, which assesses physical plausibility, reached only 47.7%, slightly below AutoDock Vina (51.2%), though still superior to other learning-based docking approaches.

A major limitation of CarsiDock was its assumption of a rigid protein structure, preventing it from capturing ligand-induced conformational changes. To address this, the research team extended its approach to cross-docking scenarios, where proteins adopt different conformations upon ligand binding. In 2025, they introduced CarsiDock-Flex [47], which incorporates receptor flexibility by refining protein pockets before docking. At the core of this new framework is CarsiInduce, an equivariant DL model designed to shift ESMFold-predicted protein structures toward holo-like conformations. CarsiDock-Flex operates in two stages. First, CarsiInduce refines the binding pocket by adjusting residue positions to better approximate the ligand-bound state, effectively mimicking the induced-fit effect. Once the binding site is refined, CarsiDock re-docks the ligand, ensuring that the final binding pose is optimized within the adjusted structure.

The introduction of CarsiInduce significantly improved binding pocket predictions. On the PoseBusters-ESMFold dataset, the model increased the fraction of accurately predicted binding pockets (RMSD $\leq $ 2.0 Å) from 71.97% to 80.81%, successfully refining 38.89% of previously mispredicted pockets. This refinement led to improved docking accuracy, with CarsiDock-Flex achieving a top-1 success rate of 56.57% (RMSD $\leq $ 2.5 Å), outperforming CarsiDock (50.25%) and reducing the average docking RMSD from 3.270 Å to 3.024 Å. These results highlight the impact of explicitly modeling protein flexibility in docking, offering a promising direction for future developments in molecular docking and structure-based drug discovery. 

### Advancements in diffusion-based flexible docking

Recent years have seen significant progress in applying diffusion models to flexible molecular docking. In this section we discuss recent advancements in diffusion-based flexible docking approaches. One advantage of diffusion models is that they, in principle, allow flexible docking to multi-chain receptors. However, since commonly used training datasets (e.g. PDBBind) are dominated by single-chain structures, recent studies [55] indicate significant performance and generalization challenges when applied to more complex multi-chain complexes.

More recently, alternative formulations such as flow matching and Schrödinger bridge methods have emerged, offering optimal transport-inspired pathways that can model “bridges” between data distributions of unbound (apo) and bound (holo) protein structures, learning vector fields, or stochastic trajectories that model conformational changes upon ligand binding. By exploiting the inherent alignment in such data, these methods capture underlying structural correspondences, leading to more accurate and physically plausible predictions of protein flexibility.

Early approaches, such as DiffBindFR [56], employed score-based diffusion techniques to predict protein–ligand complexes. Like prior methods, DiffBindFR uses an SE(3)-equivariant diffusion model to approximate the score function of the reverse denoising SDE, enabling the prediction of bound protein–ligand complexes from unbound input pairs. However, unlike previous approaches which assume rigid protein pockets, DiffBindFR jointly denoises ligand movements and pocket sidechain torsions. Trained on the PDBBind v2020 dataset, DiffBindFR achieved a docking success rate of 51.2% (RMSD $\leq $ 2 Å) and a 77.4% accuracy in recovering native-like sidechain conformations, outperforming traditional rigid docking methods.

Similar to DiffBindFR, FlexiDock [39] operates within the score-based diffusion framework but adopts a compositional approach by using two coordinated diffusion models—one for ligand flexibility and one for receptor backbone and sidechain flexibility. Inspired by the induced-fit mechanism, it refines receptor structures during inference by gradually transforming apo conformations toward holo-like states while maintaining ligand stability (Though some literature labels FlexiDock as co-folding, we classify it as flexible docking. Unlike co-folding models which are trained end-to-end to predict complexes jointly, FlexiDock uses two separately trained diffusion models for protein and ligand, combined only at inference. It also requires a pre-folded apo protein structure rather than predicting complexes directly from sequence.). Trained on PDBBind and the Saldaño apo–holo dataset, FlexiDock improved receptor RMSD accuracy from 56.58% (DiffDock) to 60.5% on PDBBind and increased receptor accuracy by over 6% on the Saldaño dataset. By preserving a physically valid ligand pose throughout the process, FlexiDock excels in cross-docking and apo-docking tasks, mitigating the performance drop observed in rigid docking methods.

Advancements in diffusion modeling introduced flow matching techniques, offering direct mappings between protein conformational states. While traditional flow matching presents an attractive option for flexible docking, its direct application can result in a complex learning task with suboptimal performance due to strict marginal constraints and the common scenario of single holo-structures for training data. To address this, FlexDock [45] proposed unbalanced flow matching as a generalization that relaxes these constraints, allowing for simpler, more learnable flows between unbound and bound states. This relaxation leads to substantial empirical improvements: on the PDBBind dataset, FlexDock increases the rate of high-accuracy protein conformation predictions (all-atom RMSD < 1 Å) from 39.8% to 44.1%, while maintaining docking accuracy comparable to other state-of-the-art models (ligand RMSD < 2 Å) and achieving faster inference speeds. On the PoseBusters benchmark, FlexDock outperforms several co-folding methods achieving a 46% docking success rate and demonstrating its generalization capability.

Building on the principles of flow matching, FlowDock [48] introduces a deep geometric flow matching model based on conditional flow matching, designed to directly map unbound (apo) protein structures to their bound (holo) counterparts. Like FlexDock, FlowDock addresses the challenges of naively training such models by developing a generalized version of UFM. This involves defining a coupling distribution $q(z)$ using apo-to-holo assessment filters (e.g. RMSD and TM-score) to measure structural similarity between unbound and bound protein structures, ensuring suitable training pairs. Representing protein–ligand complexes as geometric graphs, FlowDock refines docking predictions using a variance diminishing ordinary differential equation (VD-ODE). Integrating ESMFold-predicted structures and harmonic ligand priors, it effectively captures ligand placement and protein flexibility. This model also provides predicted structural confidence scores and binding affinity values for its generated protein–ligand complex structures, enabling fast VS. Benchmarking on the PoseBusters dataset demonstrated a 51% blind docking success rate with apo protein input structures, surpassing many generative models like single-sequence AlphaFold3. Additionally, it ranked among the top five binding affinity predictors in CASP16, highlighting its efficacy in both docking and affinity prediction tasks.

In an alternative approach to flow matching, Re-Dock [46] introduces diffusion bridge processes to jointly model ligand binding and receptor sidechain movements, emphasizing realistic induced-fit interactions. Unlike standard diffusion methods that rely on Gaussian noise perturbations, Re-Dock reformulates docking as a probabilistic induced-fit simulation, directly conditioning generative trajectories on physically valid protein–ligand interactions. This approach ensures more physically constrained docking outcomes, making Re-Dock particularly suitable for scenarios requiring high accuracy in flexible binding environments.

Collectively, these diffusion-based approaches illustrate a key advancement in molecular docking: the explicit modeling of both ligand and receptor flexibility significantly enhances docking accuracy. The field has evolved from early score-based diffusion methods—which refined ligand poses in isolation—to more integrated frameworks like DiffBindFR and FlexiDock that jointly model sidechain torsions and induced-fit effects. Flow matching techniques have further expanded the modeling capacity by learning direct, continuous mappings between unbound and bound protein states, enabling more realistic conformational transitions. However, challenges in data alignment and marginal constraints have led to the development of more robust formulations, such as Unbalanced Flow Matching, which improves learnability and generalization without sacrificing physical plausibility. Recent innovations like Re-Dock push this paradigm further by conditioning generative processes on chemically valid interactions, yielding docking predictions grounded in biophysical realism. Taken together, these advancements reflect a broader shift toward generative frameworks that unify flexibility, geometric constraints, and biochemical prior knowledge—offering a principled and scalable path toward next-generation flexible docking.

### Encoder–decoder approaches

While diffusion-based methods offer one strategy for modeling protein flexibility through iterative generative sampling, encoder-decoder architectures provide an alternative pathway. These models explicitly learn a mapping from apo protein structures and candidate ligands to refined holo-like complexes, often integrating principles from classical physics-based approaches to enhance the physical plausibility of their predictions. Below, we review two representative encoder-decoder-based methods—ApoDock [40] and FlexPose [20]—and discuss their strategies for capturing induced-fit dynamics.

ApoDock introduces a flexible docking pipeline that couples ligand-conditioned sidechain packing with classical physics-based docking to refine protein–ligand poses. In contrast to rigid docking approaches, it leverages the ApoPack module, a message-passing neural network trained on holo-apo protein pairs, to predict holo-like sidechain conformations given the ligand and backbone context. This ensures that receptor flexibility is accounted for before the ligand is placed. Once the sidechain torsion angles are predicted, classical docking tools such as Smina or Gnina use the refined protein structure to sample ligand poses. A mixture density network-based scoring function, ApoScore, then re-ranks these poses according to their physical plausibility, integrating DL predictions with traditional scoring terms. Trained on PDBBind and a curated set of apo-holo pairs, ApoDock demonstrates strong performance in recovering ligand-induced conformational changes. It achieves a top-1 docking success rate of 52% on the PoseBuster benchmark, surpassing many rigid docking approaches, and attains a 46% success rate on the Apo2Holo dataset in cases demanding extensive sidechain rearrangements.

FlexPose builds on this trend of hybrid models by directly integrating biochemical priors and geometric structure into an end-to-end EGNN. While ApoDock relies on a sequential pipeline that first adapts the receptor before docking, FlexPose takes a joint modeling approach—encoding both protein and ligand atoms as nodes in a geometric graph and refining their conformations simultaneously through iterative message passing. To improve its handling of diverse conformations, FlexPose undergoes conformation-aware pre-training across a broad chemical space and incorporates low-confidence docking poses during training to enhance robustness. These strategies allow it to achieve a 64.8% success rate in predicting ligand binding poses using apo protein structures on APObind and PDBBind, significantly outperforming rigid docking methods. Additionally, it demonstrates a 70.5% success rate in cross-docking, highlighting its ability to model induced-fit interactions that rigid docking approaches often fail to capture.

## Co-folding approaches

### Sequence to structure, UMOL, and CASP 15

The development of UMOL [49] and the recent inclusion of a sequence based ligand docking category in CASP15 mark a significant paradigm shift in molecular docking. This emerging class of methods, known as co-folding approaches, predicts the structure of protein–ligand complexes as a single task. Co-folding models like UMOL directly predict fully flexible, all-atom protein–ligand complexes from sequence, capturing both sidechain and backbone flexibility, without requiring predefined binding sites or template structures. This makes them especially well suited for de novo drug discovery and novel targets.

UMOL extends AlphaFold2’s EvoFormer architecture to jointly model protein–ligand interactions. The method processes the protein through multiple sequence alignment (MSA) features and encodes the ligand using a bond matrix derived from its SMILES representation. These are jointly embedded and passed through 48 EvoFormer blocks, enabling cross-communication between the protein and ligand representations. A 3D structure module then refines atomic coordinates through iterative updates, producing protein–ligand complexes that account for mutual flexibility. It also provides plDDT-based confidence scores that correlate with binding affinity, allowing it to distinguish strong binders from weak ones—a critical feature for early-stage drug discovery.

Additionally, the recent introduction of a sequence-based docking category in CASP15 underscores the field’s growing shift toward sequence-to-structure modeling. Recent DL models—such as Chai-1 and AlphaFold3—have extended this framework beyond protein–ligand prediction to encompass a broader range of biomolecular assemblies, including protein–nucleic acid and protein–protein complexes. The following section explores these models in greater detail, along with their current limitations.

### Multi-modal foundation models

The emergence of sequence to structure models has driven the development of multi-modal foundation models such as AlphaFold3 [36] and RoseTTAFold All-Atom [50]. These co-folding models exhibit remarkable abilities in predicting a broad spectrum of biomolecular interactions, including protein–protein, protein–DNA, protein–RNA, and protein–ligand. Notably, their general-purpose nature does not compromise performance on specialized tasks like protein–ligand docking; rather, they achieve state-of-the-art performance across multiple protein–ligand benchmarks. This advantage likely stems from their extensive training on diverse biomolecular data, allowing them to learn fundamental principles of biomolecular interactions that transcend any single modality.

RFAA extends the RoseTTAFold2 backbone by incorporating a hybrid representation: one-dimensional sequence data, two-dimensional atomic graphs capturing covalent connectivity, and three-dimensional spatial features. It replaces generative diffusion components with a deterministic, regression-based architecture, using template-matching layers and a per-atom Frame Aligned Point Error loss function to enforce geometric consistency. This allows the model to directly refine atomic coordinates over successive layers. RFAA outperformed traditional docking tools such as AutoDock Vina in blind benchmarks like CAMEO and PoseBusters, achieving a 42% success rate (ligand RMSD < 2.0 Å) demonstrating its generalization ability to novel protein and ligand clusters. Nevertheless, its reliance on generative mechanisms occasionally led to misassignment of ligand chirality and limited specificity in flexible binding pockets.

AlphaFold3 (AF3) represents a significant advancement over prior models like RFAA by integrating a diffusion-based structure module that iteratively refines atomic coordinates across multiple spatial scales. This generative approach enables AF3 to more accurately capture induced fit effects. AF3 also replaces the evoformer module used in AlphaFold2 with a pairformer module, which enhances the representation of pairwise atomic interactions and reduces reliance on multiple sequence alignments. By removing fixed stereochemical constraints and predefined bonding rules, AF3 increases its adaptability to a broader range of molecular types and chemical environments. On the PoseBusters benchmark, it achieved a 80% success rate (ligand RMSD < 2.0 Å), outperforming RFAA and traditional docking tools like AutoDock Vina, particularly in flexible and chemically diverse binding scenarios. However, its generative nature occasionally produced non-physical ligand conformations and stereochemical inaccuracies, requiring post-processing to ensure structural plausibility.

Chai-1 [51] built upon AF3’s architecture and training strategy, introducing the ability to be prompted with constraint features such as pocket conditioning and docking to apo structures, thereby enhancing prediction accuracy. During training, these constraints were randomly sampled from ground-truth structures using chain-wise and token-wise dropout, promoting robust predictions even with partial or noisy experimental data. On the PoseBusters benchmark, Chai-1 achieved a 81% success rate (ligand RMSD < 2 Å), slightly surpassing AF3. Incorporating explicit docking constraints further increased accuracy to 85.5%. Chai-1 also performed strongly on the CASP15 monomer dataset, attaining a C$\alpha $-LDDT of 0.849. However, like AF3, it remained susceptible to occasional ligand chirality errors.

Despite recent progress, many models remained closed-source, limiting transparency and collaboration. Boltz-1 [52] tackled this by introducing the first open-source DL model for biomolecular interaction modeling, matching the performance of AF3 and Chai-1. Like Chai-1, it supports both blind and pocket-specific docking. While based on the AF3 framework, Boltz-1 introduced key changes to improve training and structural accuracy—such as refining its MSA module, stabilizing transformer layers, and integrating a confidence model directly into the trunk. A Kabsch alignment step further improved structural output, and optimizations like chunked attention enhanced efficiency. Benchmarking showed Boltz-1 performing competitively in docking and structure prediction. On CASP15, it achieved a median C$\alpha $-LDDT of 0.849. In the PoseBusters benchmark, it kept ligand RMSD below 2 Å, capturing ligand flexibility and induced fit effects better than traditional methods. Though it still faced issues common to generative models—like stereochemical errors and occasional chain overlaps—its open-source design enabled ongoing improvements, making it a transparent and scalable alternative to proprietary tools.

The latest addition to the co-folding paradigm, NeuralPLexer3 (NP3) [53] introduces a flow-based generative modeling framework for more efficient and physically realistic prediction of biomolecular complex structures. Unlike previous score-based diffusion models like AF3, NP3 leverages continuous normalizing flows (CNFs) and flow matching to generate atom-level accurate structures while significantly reducing computational overhead. Additionally, NP3 incorporated physics-informed priors and optimal transport symmetry correction, ensuring that protein–ligand interactions were modeled with greater structural accuracy and faster inference times. On the PoseBusters benchmark with PB-Validity checks, NP3 achieved a 77.9% success rate, surpassing AF3’s 73.1% and significantly outperforming traditional docking methods like Vina (59.7%) and GOLD (58.1%). NP3 also outperformed AF3 on the NPBench evaluation, particularly in modeling covalent ligand interactions and flexible protein–ligand complexes.

### Limitations of co-folding approaches

Despite their impressive performance and features such as native support for multi-chain inputs and broader biomolecular interaction prediction, co-folding models like AlphaFold3 (AF3) and Chai-1 share a fundamental limitation common to many DL approaches in their limited ability to enforce core physical and chemical principles. While these models achieve high structural accuracy, recent studies [55, 57, 58] suggest that they rely more on learned statistical patterns rather than on true molecular interaction principles. Masters et al. (2024) [58] demonstrated that AF3 continues to predict nearly identical ligand poses even when critical binding residues are mutated—whether by removing key sidechain interactions, crowding the binding pocket, or introducing repulsive residues. This suggests that rather than predicting binding interactions based on an underlying physical energy landscape, current co-folding models primarily follow global structural motifs learned from their training data. Furthermore, they found that AF3 frequently assigns high-confidence scores to physically implausible structures, indicating a lack of self-correction mechanisms. Additionally, they found that co-folding models also perform poorly in multi-ligand environments, often generating steric clashes and unrealistic ligand placements, again suggesting that they do not learn the physical principles of biomolecular interactions.

Additionally, co-folding approaches have been criticized for overfitting to training data, excelling on familiar protein–ligand complexes but failing on novel targets. Morehead et al. [55] found that while DL co-folding methods outperform traditional docking tools in standard benchmarks, they struggle to balance structural accuracy with chemical specificity, especially when confronted with novel protein sequences or uncommon binding sites. Additionally, they argue that AF3’s dependence on MSAs—which enhances accuracy for well-represented proteins—leads to performance degradation when evolutionary data is unavailable. In contrast, Chai-1, which integrates protein language models with MSAs, showed a more balanced performance, however, still struggled with out-of-distribution data. They also found that co-folding models struggle with under-represented protein classes, such as immune system and metal-transport proteins, revealing a bias toward well-characterized interactions. Collectively, these findings suggest that while co-folding models perform well within the scope of their training data, their generalizability remains a significant challenge.

While co-folding models represent a significant advancement, their limitations highlight the need for stronger physical constraints, improved generalization to novel proteins, and better modeling of multi-ligand interactions. Further research is required to understand these shortcomings and develop solutions, ensuring that co-folding methods can be reliably applied in drug discovery and *de-novo* drug design.

## Future directions

Having reviewed the evolution of DL methods in molecular docking—from the early rigid-body models to ligand-flexible and finally, fully flexible approaches—it is evident that these methods have dramatically advanced our ability to predict protein–ligand interactions. However, despite these advancements, several challenges persist.

Earlier studies, such as PoseBusters, highlighted the challenge of generalizing to proteins structurally distinct from those in the training data. Recent protein-flexible models have improved upon earlier rigid models by accounting for conformational changes in protein pockets, preventing the model from merely memorizing pocket configurations and thereby enhancing robustness and generalizability. While these models show promise in predicting interactions for unseen proteins, they still struggle with proteins that differ significantly from their training data. Beyond simply increasing dataset size and diversity, a promising approach—drawing inspiration from multi-biomolecular co-folding models—is to expand datasets beyond protein–ligand complexes to include interactions such as protein–protein, protein-DNA, and protein-RNA. This broader diversity may enable models to learn fundamental interaction principles rather than relying on pattern recognition.

From a practical standpoint, a promising strategy may be to analyze these models to identify the types of proteins they perform well on versus those they struggle with, allowing for more informed application. Analogous to mixture of experts in large reasoning models, a potential next step could involve splitting these models into smaller specialized “expert” models, fine-tuned neural networks designed for specific protein families to improve generalization and reliability.

Another common limitation of molecular docking models is their inability to adhere to strict physical laws which guide protein–ligand docking. Diffusion-based molecular docking programs, for example, frequently struggle with predicting physically implausible structures, generating unrealistic structures at a much higher rate than traditional docking methods that are based on physical simulations or energy calculations. To enhance physical plausibility, integrating principles from traditional physics-based methods, such as learning a funneled energy landscape [22], seems highly promising, particularly in more difficult docking scenarios like multi-ligand environments where current methods still frequently predict steric clashes. A complementary approach involves training models on molecular dynamics simulation data, which, although slow to generate, offers a rich source of physically grounded trajectories. An emerging research direction is the use of flow matching models to learn and approximate molecular dynamics trajectories [59]. These models aim to capture continuous, physically plausible transitions between molecular conformational states while substantially reducing the computational cost and time required to perform full-scale molecular dynamics simulations.

Docking in more challenging and biologically realistic scenarios remains a critical but underexplored frontier. Current methods are often benchmarked under simplified conditions, such as single-chain targets, idealized binding sites, and neglect of key environmental factors like solvent effects, metal ions, and cofactors. These limitations restrict the applicability of docking models to real-world systems. In particular, the performance of DL-based methods on multi-chain protein receptors remains poorly characterized, despite their significance in many practical applications. To identify potential limitations and guide future improvements, systematic investigation of model behavior in these more complex settings is essential. Addressing these gaps will require both the development of docking models that can handle such complexity and the design of more rigorous, biophysically meaningful evaluation metrics. Improved benchmarking in these more difficult settings is essential for assessing generalization and advancing the field.

For VS applications, balancing structural accuracy with computational efficiency remains a key challenge. Flexible docking methods provide a more favorable trade-off between speed, accuracy, and physical realism for high-throughput VS workflows. Although co-folding approaches can predict complexes without requiring predefined structures, their long inference times currently limit their practicality in large-scale VS. A potential solution may lie in model distillation, reducing the size and computational overhead of these models while preserving performance. Additionally, the introduction of more open-source models will likely allow researchers to more easily analyze and improve core components of models, such as the Pairformer, or diffusion module, which may further enhance both efficiency and accuracy.

**Key Points**

- DL has transformed molecular docking, enabling faster and more accurate predictions than traditional methods while enabling more challenging tasks such as blind docking.
- Many traditional docking models assume rigid protein structures, but this simplification limits real-world applicability—especially in apo and cross-docking scenarios where proteins undergo significant conformational changes. Recent DL approaches have made notable progress in modeling protein flexibility, addressing a long-standing limitation of previous methods.
- This review categorizes recent flexible docking methods into implicit and explicit models, as well as flexible docking and co-folding strategies. It highlights key examples and explains how each method incorporates protein flexibility into their predictions.
- Diffusion-based generative models have emerged as powerful tools for modeling protein–ligand interactions. Techniques such as geometric flow matching and Schrödinger bridges enable models to learn transitions between unbound and bound protein states. These approaches are also central to many co-folding methods, which predict protein–ligand complexes directly from sequence data.
- Key challenges remain, including limited generalization to novel proteins, the generation of physically implausible structures, and reliance on weak evaluation metrics like RMSD. Future work should emphasize physically grounded modeling, improved validation criteria, and broader generalization through physics-informed machine learning.

Conflict of interest: None declared.

## Data Availability

No new data were generated or analyzed in support of this study.
